# Hospital-reported Pneumococcal Susceptibility to Penicillin^1^

**DOI:** 10.3201/eid1001.030140

**Published:** 2004-01

**Authors:** Joshua P. Metlay, Charles C. Branas, Neil O. Fishman

**Affiliations:** * VA Medical Center, Philadelphia, Pennsylvania, USA; University of Pennsylvania, Philadelphia, Pennsylvania, USA

**Keywords:** drug resistance, streptococcus pneumoniae, microbial sensitivity tests

## Abstract

Geographic variation in drug susceptibility among isolates of *Streptococcus pneumoniae* has influenced national treatment guidelines for community-acquired pneumonia. Whether individual hospital susceptibility data provide reliable and valid information for providers is unclear. We examined the geographic and temporal variability in hospital-reported rates of pneumococcal susceptibility. We surveyed all 52 hospitals that provided acute adult care in the five counties surrounding Philadelphia and collected data on levels of penicillin susceptibility among all pneumococcal blood isolates from 1998 to 2000. In 1998, pneumococcal nonsusceptibility to penicillin varied from 0% to 67% of all blood isolates across the 33 hospitals with >10 isolates in that year. Hospital location did not correlate with the level of reported pneumococcal susceptibility (p = 0.8). In addition, correlations were not significant in reported pneumococcal susceptibility to penicillin within individual hospitals during the 3 years.

Antimicrobial drug treatment of patients with community-acquired pneumonia is largely empirical because the pathogen is not frequently identified. Even when a bacterial pathogen is identified, antimicrobial drug susceptibility information is frequently delayed for a few days, necessitating empiric treatment decisions. Guidelines for empirical treatment have been provided by several organizations, emphasizing the selection of agents based on the probability of specific pathogens in different clinical settings (*1*–*3*). One consistent recommendation of all guidelines for all clinical settings is that antimicrobial therapy cover *Streptococcus pneumoniae* because it is the most common bacterial pathogen and among the most virulent.

A number of factors guide the selection of empirical therapy for patients with community-acquired pneumonia, including drug efficacy, side effect profile, and ease of administration. In addition, the recent emergence of antimicrobial drug resistance among clinical isolates of *S. pneumoniae* has raised considerable concern that a number of drugs historically endorsed by national treatment guidelines may no longer be effective. The recommendations to avoid β-lactams and macrolides in high-risk settings has not been preceded by clinical studies demonstrating that drug resistance in vitro translates into clinical treatment failures (*4*). Regardless, emerging resistance to penicillins, tetracyclines, and macrolides has prompted newer versions of treatment guidelines to recommend abandoning these therapies in favor of newer therapies, such as fluoroquinolones, in settings where the risk for a drug-resistant pneumococcal infection is high. Given that penicillin-resistant *S. pneumoniae* are increasingly multidrug resistant (*5*), if the risk for penicillin-resistant *S. pneumoniae* is high, the risk for resistance to macrolides and tetracyclines is correspondingly high.

Specific risk factors for infection with penicillin-resistant *S. pneumoniae* have not been clearly identified. Individual risk factors include prior exposure to antibiotics and exposure to young children in daycare (*6*), although no specific rules guide the interpretation of these risk factors. In addition, awareness of substantial geographic variation in the proportion of drug-resistant *S. pneumoniae* has lead some groups to recommend consideration of regional susceptibility patterns in the choice of empirical therapy for community-acquired pneumonia (*2*). Unfortunately, routine isolation of *S. pneumoniae* is highly dependent on the clinical setting. For example, blood and sputum samples are rarely sent from outpatient settings (*7*); thus, local rates of pneumococcal drug resistance are primarily determined by the phenotypes of bacteria isolated from sicker patients who require hospitalization. Whether results from local hospital microbiology laboratories provide valid information for guiding treatment is unclear.

The specific aims of this study were to compare the rates of penicillin resistance among pneumococcal blood isolates across all acute-care hospitals within a five-county region of eastern Pennsylvania. We examined the relationship between pneumococcal resistance, rates of pneumococcal isolation, and geographic location to determine the reliability of individual hospital rates for guiding treatment decisions. Our underlying hypothesis was that individual hospital rates of penicillin resistance among clinical isolates of *S. pneumoniae* would be poorly correlated across time and space, emphasizing the inherent dangers in using individual hospital rates to guide empirical treatment decisions in the community.

## Methods

### Design

This study was a cross-sectional study of penicillin susceptibility among clinical blood isolates reported at all acute-care hospitals in the Delaware Valley Case Control Network (DVCCN). DVCCN is a population-based network of all adult acute-care hospitals in the five Pennsylvania counties around the metropolitan Philadelphia region. These counties (Bucks, Montgomery, Chester, Delaware, and Philadelphia) contain 52 adult acute-care hospitals serving a population of 3.4 million residents.

### Data Collection

Data were collected during two periods. During the first data collection, microbiology laboratory supervisors were contacted by telephone at each area hospital during January 1999 to collect data on all pneumococcal blood isolates during the preceding year. Local laboratory personnel reported cumulative numbers of pneumococcal blood isolates and the proportion of isolates that were penicillin nonsusceptible for the 12-month period. When available, laboratories separately reported susceptibility percentiles as susceptible, intermediate susceptible, and resistant; otherwise, the laboratories reported the combined percentile nonsusceptible (intermediate plus resistant). We collected information on unique isolates but did not confirm that these represented unique episodes of patient infections. However, we think hospitals generally reported only one isolate per patient because guidelines recommend that hospitals report cumulative susceptibility data on the basis of single isolates per patient per reporting period (*8*).

A second data collection was conducted in early 2001. The same hospital laboratories were contacted by mail with follow-up telephone calls requesting the same information on cumulative numbers of pneumococcal blood isolates and the proportion of isolates that were penicillin nonsusceptible for the 12-month period. Data were requested for 1999 and 2000 separately, if available.

### Analysis

Annual number of pneumococcal blood isolates and penicillin-susceptibility rates were summarized for each hospital for each year. Standard National Committee for Clinical Laboratory Standards (NCCLS) criteria define three categories of penicillin susceptibility for *S. pneumoniae*: susceptible (MIC <0.1 μg/mL), intermediate susceptible (MIC 0.1–1.0 μg/mL) and resistant (MIC >2.0 μg/mL) (*9*). Penicillin-nonsusceptible isolates are defined as intermediate susceptible and resistant isolates combined. For all analyses, we examined the proportion of isolates that were nonsusceptible. We specifically excluded any hospital site reporting <10 isolates per year as too unreliable to provide point estimates of resistance rates. This level is consistent with NCCLS guidelines recommending hospital laboratories report cumulative susceptibility data only for organisms for which there is a minimum of 10 isolates per reporting period (*8*). Descriptive statistics were developed for the distributions of susceptibilities and annual pneumococcal isolation rates. Similar to prior studies of hospital-level variation (*10*,*11*), we report the proportion of hospitals with >5% deviation from the overall regional mean rate of resistance because lower levels of deviation are unlikely to influence prescribing decisions.

To analyze the reliability of individual hospital rates of pneumococcal resistance, we examined the correlation of rates across each of the 3 years of data collection. We restricted these analyses to those hospitals reporting >10 pneumococcal blood isolates in 1998, 1999, and 2000. Correlations between any 2 years were calculated with Spearman’s correlation coefficient, testing the assumption that pneumococcal susceptibility levels at individual hospitals should be correlated year to year, regardless of whether overall susceptibility levels for the region were rising, falling, or remaining constant. In addition, we calculated an intra-class correlation coefficient across all 3 years of data at the hospital level by using a general linear model for analysis of variance (*12*).

Next, we tested the assumption that the proportion of nonsusceptible *S. pneumoniae* at each hospital should demonstrate an underlying geographic clustering. We tested the geographic clustering of pneumococcal resistance rates with two methods. First, we analyzed whether the location of each hospital at the county level was associated with the proportion of nonsusceptible pneumococci by using the nonparametric Kruskal-Wallis test. Second, we conducted Geographic Information System (GIS) mapping to visually judge the spatial distribution of hospital susceptibility rates. Hospitals were geographically assigned longitude and latitude coordinates on the basis of the hospital address by using GIS mapping software (Maptitude, Caliper Corporation, Newton, MA) to display the proportion of nonsusceptible pneumococci at each hospital location on the map. Using data from 1998 only, we graphically represented the proportion of nonsusceptible pneumococci as a proportional symbol map (bubble plot), where each hospital location is represented by a circle whose radius is proportional to the level of penicillin nonsusceptibility among pneumococcal blood isolates.

We tested the null hypothesis that no spatial autocorrelation of the nonsusceptibility rates occurred with Moran’s I statistic, based on a proximity matrix calculated from the city-block distances between the longitude-latitude coordinate points of hospital addresses. Moran’s I is a spatial cross-product coefficient that is interpreted similarly to the Pearson correlation coefficient (*13*,*14*).

## Results

For the survey of 1998 susceptibility results, we received responses from 47 of 52 hospitals in the DVCCN. However, in two instances, the results from multiple hospitals were combined at a single laboratory and could not be separated for reporting purposes. Of the 45 laboratories reporting results, 33 laboratories reported >10 pneumococcal blood isolates during 1998. Among the 33 sites, the median number of pneumococcal blood isolates during 1998 was 19 (range 10-92).

**Figure 1:**
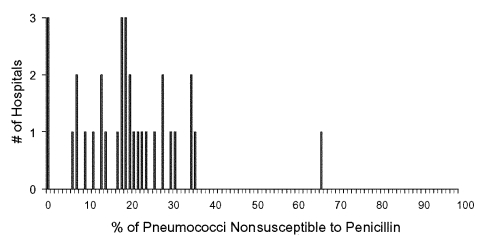
Proportion of penicillin-nonsusceptible pneumococcal bloodstream infections at hospitals in the Delaware Valley. The number of hospitals with each reported level of penicillin nonsusceptibility among all pneumococcal bloodstream isolates at each hospital in 1998 are shown. Penicillin nonsusceptibility was defined as any isolate with a penicillin MIC >0.1 μg/mL. Hospitals with <10 isolates in 1998 were excluded.

Figure 1 displays the frequency distribution of the proportion of isolates with nonsusceptibility to penicillin as reported by each hospital in 1998. The proportion of nonsusceptible isolates ranged from 0% to 67%, with a mean proportion of 21%. Sixty-one percent of hospitals were >5% above or below the mean. If we only included hospitals with >20 pneumococcal isolates in 1998, a total of 16 hospitals would be included in the analysis; the proportion of nonsusceptible isolates ranged from 0% to 36%, with a mean proportion of 20%. Fifty-six percent of hospitals were >5% above or below the mean proportion of nonsusceptible isolates.

For the 1999 and 2000 survey results, we received responses from 31 hospital laboratories. Of these, 23 hospital laboratories reported >10 pneumococcal isolates in 1 of the 2 study years. The mean proportion of isolates with reduced susceptibility to penicillin was 19% in 1999 and 24% in 2000. Fifty-three percent of sites were >5% above or below the mean in 1999, and 65% of sites were >5% above or below the mean in 2000.

The Table summarizes the relationship between each hospital’s annual proportion of nonsusceptible pneumococcal blood isolates during the 3 years of the study. For hospitals reporting >10 isolates in 1998, 1999, and 2000 (N = 15), the proportion of nonsusceptible isolates at each hospital was poorly correlated between any 2 years. For 1998 to 1999, the Spearman correlation coefficient was –0.07 (p = 0.82), for 1999 to 2000, the Spearman correlation coefficient was 0.28 (p = 0.32), and for 1998 to 2000, the Spearman correlation coefficient was –0.03 (p = 0.91). Across all 3 years, the overall intraclass correlation coefficient was 0.18.

**Table:** Annual variation in proportion of penicillin-nonsusceptible isolates at hospitals in the Delaware Valley

Site	Annual no. blood isolates			% penicillin nonsusceptible		
	1998	1999	2000	1998	1999	2000
A	25	21	26	0	33	27
B	12	15	17	0	13	29
C	29	25	29	24	20	28
D	14	18	12	14	22	8
E	44	39	28	18	23	25
F	27	19	20	19	21	20
G	26	56	35	19	23	17
H	62	15	22	19	0	27
I	10	13	12	20	38	50
J	44	15	32	20	33	16
K	92	45	87	22	4	8
L	35	31	30	29	16	20
M	41	36	34	29	14	38
N	33	32	35	36	13	9
O	19	15	19	37	40	47

**Figure 2:**
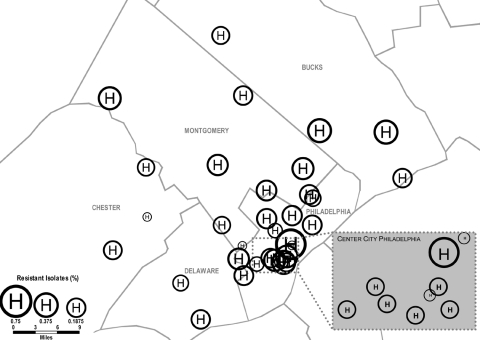
Geographic distribution of penicillin nonsusceptibility among pneumococcal isolates at 33 hospitals in the Delaware Valley in 1998. The figure is a proportional symbol map (bubble plot). Each hospital location is represented by a circle with an H in the center at the corresponding longitude and latitude of the hospital. The radius of the circle is directly proportional to the proportion of penicillin-nonsusceptible pneumococci at each hospital in 1998. The range is 0% to 67%. Hospitals with <10 isolates in 1998 were excluded. An insert magnifies the geographic distribution of hospitals clustered in the center of Philadelphia.

Location of hospital by county did not correlate with the proportion of pneumococcal blood isolates nonsusceptible to penicillin (p = 0.45, 0.37, 0.08 in 1998, 1999, and 2000, respectively). Figure 2 is a proportional symbol map with each hospital location represented by a circle whose radius corresponds to the proportion of nonsusceptible pneumococcal blood isolates at each hospital in 1998. Overall, Figure 2 shows geographic clustering of the 33 hospitals reporting >10 pneumococcal blood isolates in 1998, 14 of which are gathered in Philadelphia, the most heavily populated county in our five-county study area. No geographic clustering in the proportion of nonsusceptible pneumococci isolated at each hospital was evident from our data. This finding is supported by a Moran correlation coefficient of –0.01, demonstrating a lack of statistically significant spatial autocorrelation in the proportion of nonsusceptible hospital pneumococci at hospitals across the five-county region (p = 0.80).

## Discussion

Pneumococcal drug resistance has increased at a fast rate. Most resistant isolates now demonstrate reduced susceptibility to multiple antimicrobial drug classes, including β-lactams, macrolides, and tetracyclines. However, substantial geographic variability in the proportion of pneumococci resistant to different antimicrobial drugs at the international and national levels suggests that the impact of resistance on empirical treatment decisions may vary across regions. Indeed, at least one guideline recommends that the selection of empirical therapy for outpatients with community-acquired pneumonia should be influenced by regional antimicrobial drug susceptibility patterns for *S. pneumoniae* (*2*).

For most physicians, the obvious source of information on community-acquired pneumonia is the microbiologic susceptibility results from their local hospital laboratory. Hospitals frequently provide this information in the form of antibiograms, specifically designed to aid in antimicrobial drug selection. The validity of this information is largely unknown. This study demonstrates that substantial variability exists in hospital-reported rates of pneumococcal drug resistance over a small geographic region and that the rates at each site are poorly correlated over time. In addition, using a variety of approaches, we were unable to demonstrate substantial geographic clustering of the data at the individual hospital level, suggesting that the individual hospital rates poorly reflect an underlying rate of resistance in the community.

Other studies have demonstrated similar heterogeneity in the proportion of drug-resistant pneumococci reported by hospitals over small geographic areas. The Centers for Disease Control and Prevention reported levels of pneumococcal resistance to penicillin among invasive isolates from all hospitals within the seven regions in the United States that comprise their Active Bacterial Core Surveillance program. The proportion of penicillin-nonsusceptible *S. pneumoniae* ranged from 15.3% to 38.3% across the seven regions. The proportion of hospitals within each region that were >5% below or above the average for the region ranged from 35% to 76%. No hospital characteristics, including proportion of isolates from children or from black patients, predicted deviation from the regional average (*11*). More recently, 16 hospitals in Brooklyn, New York, participated in boroughwide surveillance for *S. pneumoniae* in 1997 and 1999. Aggregating the hospitals into the western and eastern ends of the borough demonstrated a significant difference in the proportion of penicillin-susceptible isolates, 57% versus 75% (p = 0.046) (*15*).

Our hypothesis that individual hospital rates of drug-resistant *S. pneumoniae* should demonstrate geographic clustering is based on the assumption that rates of resistance should reflect the underlying distribution of drug resistance in the source community. Prior research has demonstrated that patients with community-acquired pneumonia are hospitalized on average <5 miles from their place of residence (*16*). Thus, hospitals within the same geographic region should admit patients with similar underlying rates of drug resistance. Moreover, if the pneumococcal resistance rates reported by individual hospitals should be used to guide physician antibiotic prescribing decisions for patients with community-acquired pneumococcal infections, hospitals serving similar communities should report similar rates of resistance.

Beyond reflecting true variability in the underlying community levels of drug resistance, hospital-level variation in the proportion of penicillin-susceptible pneumococcal isolates reflects at least four phenomena. First, chance error can create significant variability in hospital-level results particularly since many hospitals have only a small number of invasive pneumococcal isolates per year. We excluded hospitals with <10 isolates in each study year from our analyses to reduce the role of chance in our findings, but the relatively small number of isolates at each site undoubtedly contributed to the significant year-to-year variability and lack of geographic clustering. However, a minimum of 10 isolates per year is the NCCLS-recommended minimum number of isolates for reporting cumulative antimicrobial susceptibility results for any species in any reporting period (*8*).

Second, variation in testing strategies at individual hospitals may create bias in the proportion of isolates identified as nonsusceptible if the thresholds for sending microbiologic tests vary according to characteristics that are likely to influence the probability of a drug-resistant infection. While this may be an important determinant of variability in drug-susceptibility rates among clinical isolates from respiratory and sinus samples, we believe that it is less likely to explain variability in susceptibility rates among blood isolates since thresholds for sending blood cultures are more standardized across clinical practices, particularly in the management of patients hospitalized with community-acquired pneumonia.

Third, hospitals may differ in their methodologic approach and quality assurance for pneumococcal susceptibility testing. However, a study based on a nationwide College of American Pathologists proficiency survey of hospital laboratories demonstrated that, while the use of specific susceptibility tests varies substantially, few major interpretive errors occurred in assigning the susceptibility to standardized pneumococcal isolates (*17*). In addition, prior studies have found that the results of susceptibility data generated at local hospital facilities provide a reasonable surrogate for susceptibility data generated at centralized facilities (*18*).

Finally, geographic proximity of hospitals does not ensure that the patient populations served by those hospitals come from the same communities. Indeed, substantial literature has established important variation in hospital referral patterns that creates diversity in the source of patient populations among geographically proximate hospitals. Future research will need to consider the role of referral patterns in explaining some of the hospital-level variability observed in this and other studies.

One limitation of this study is our focus on a single geographic region in eastern Pennsylvania. Patterns and determinants of hospital-level variation in pneumococcal drug susceptibility may produce different results in other regions. In addition, because we focused on hospitals providing adult care, we cannot generalize our results to pediatric hospitals.

Regardless of whether individual hospitals provide valid information on local levels of pneumococcal susceptibility, variability in these levels is only clinically meaningful if the range of susceptibility crosses some threshold for the empirical choice of specific antimicrobial drugs. Currently in the United States, empirical use of penicillins for the treatment of community-acquired pneumonia is uncommon relative to the use of fluoroquinolones and macrolides (*19*). However, as resistance to these classes of antimicrobial drugs continues to grow, physicians will need to determine appropriate thresholds for switching to yet newer antimicrobial agents. In these settings, valid information on local rates of pneumococcal drug susceptibility will become increasingly important. Ideally, data should be derived from large samples reflective of the region for which the empirical recommendations apply*.* Continued research is needed to determine whether susceptibility data provided by local hospital microbiology laboratories can ever serve this purpose.
